# An *Arabidopsis* Cytokinin-Modifying Glycosyltransferase UGT76C2 Improves Drought and Salt Tolerance in Rice

**DOI:** 10.3389/fpls.2020.560696

**Published:** 2020-11-05

**Authors:** Yanjie Li, Fangfei Liu, Pan Li, Ting Wang, Chengchao Zheng, Bingkai Hou

**Affiliations:** ^1^The Key Laboratory of Plant Development and Environment Adaptation Biology, Ministry of Education, Shandong University, Qingdao, China; ^2^School of Life Sciences, Shandong University, Qingdao, China; ^3^College of Pharmacy>, Liaocheng University, Liaocheng, China; ^4^State Key Laboratory of Crop Biology, College of Life Sciences, Shandong Agricultural University, Taian, China

**Keywords:** abscisic acid, *AtUGT76C2*, drought stress, salt stress, glycosyltransferase, rice

## Abstract

Drought and salt stresses are common environmental threats that negatively affect rice development and yield. Here we report that the overexpression of *AtUGT76C2*, a cytokinin glycosyltransferase, in rice modulates cytokinin homeostasis and confers the plants an eminent property in drought and salt tolerance. The transgenic plants exhibit sensitivity to salt and drought stress as well as abscisic acid during the germination stage and the postgermination stage while showing enhanced tolerance to drought and salinity at the young seedling stage and the mature stage. The overexpression of *UGT76C2* decreases the endogenous cytokinin level and enhances root growth, which greatly contributes to stress adaptation. In addition, the transgenic plants also show enhanced ROS scavenging activity, reduced ion leakage under salt stress, smaller stomatal opening, and more proline and soluble sugar accumulation, which demonstrate that UGT76C2 acts as an important player in abiotic stress response in rice. To explore the molecular mechanism of UGT76C2 in response to stress adaptation, the expressions of eight stress-responsive genes including *OsSOS1*, *OsPIP2.1*, *OsDREB2A*, *OsCOIN*, *OsABF2*, *OsRAB16*, *OsP5CR*, and *OsP5CS1* were detected, which showed notable upregulation in *UGT76C2* overexpression plants under salt and drought stresses. Our results reveal that the ectopic expression of *AtUGT76C2* confers the transgenic rice many traits in improving drought and salt stress tolerance in both developmental and physiological levels. It is believed that *AtUGT76C2* could be a promising candidate gene for cultivating saline- and drought-tolerant rice.

## Introduction

Drought and salinity are the most common abiotic threats limiting plant growth and crop yield. As the most consumed staple food in the world, rice is extremely sensitive to these stresses. Rice requires sufficient water during the whole life cycle, especially at the grain filling stage. Until now, drought and salinity have become major factors causing rice yield loss. Thus, a study exploring critical saline- and drought-tolerant genes as well as revealing their molecular mechanisms is a critical prerequisite for cultivating saline- and drought-tolerant rice *via* molecular design breeding.

Until now, many stress-regulated genes have been identified to improve stress resistance in plants, which can be grouped into four types: genes involved in the synthesis of osmotic regulators, such as proline biosynthesis gene *OsP5CS1* and *OsP5CR* (Hu et al., 1992; Sripinyowanich et al., 2013); genes involved in ion transportation, such as *SOS1* (Shi et al., 2000); antioxidant-related genes, such as *CAT* (catalase), *APX* (ascorbate peroxidase), and *SOD* (superoxide dismutase) (Mhamdi et al., 2010; Verma et al., 2019); and genes regulating signaling cascades, such as protein kinases (Kang et al., 2017), transcription factors, and so on (Xiong et al., 2014; Chen et al., 2015). However, to enrich the present knowledge and uncover the molecular mechanism on stress regulation in every aspect, more pathways still need to be elucidated.

Cytokinins (CK) are N6-substituted adenine derivatives which are originally defined as a key regulator participating in various plant developmental activities, including organ formation, apical dominance, and leaf senescence (El-Showk et al., 2013). Nowadays, increasing evidence also indicates the action of cytokinin in response to stresses (Hai et al., 2020). A recent cytokinin-responsive transcriptome analysis in rice revealed that a large number of genes are involved in both biotic and abiotic stresses (Raines et al., 2016). It is also reported that cytokinin is required for responding to a series of environmental factors including temperature, drought, osmotic stress, salinity, nutrient stress, plant pathogens, and herbivores (Raines et al., 2016; Cortleven et al., 2019). Genetic studies by modifying cytokinin level in plants indicate that it generally serves as a negative player in stress response (Nishiyama et al., 2011). For instance, the overexpression of the cytokinin-degrading enzyme cytokinin oxidase/dehydrogenase gene enhanced the drought and heat stress tolerance in transgenic tobacco plants (Macková et al., 2013). Previous studies found that AHK2, AHK3, and AHK4 are negative regulators of dehydration and salt tolerance, and the *ahk2* and *ahk3* mutants displayed higher survival rates under severe water deprivation (Tran et al., 2007; Kang et al., 2012). However, some studies found that cytokinin could also function as a positive regulator in drought stress adaptation (Hai et al., 2020). For example, the ectopic expression of the isopentenyltransferase gene (*IPT*) that encodes a rate-limiting enzyme in cytokinin biosynthesis increases endogenous cytokinin levels as well as improves drought stress tolerance in transgenic cotton (Kuppu et al., 2013), creeping bentgrass (Xu et al., 2016), eggplant (Xiao et al., 2017), and tropical maize (Leta et al., 2016). It might be attributed to the choice of promoter, which led to the regulation of different pathways.

Plants tend to fine-tune the developmental process and environmental responses *via* crosstalk between phytohormones. A number of studies have revealed the interplay between cytokinin and abscisic acid (ABA), which act antagonistically in regulating stress response. In contrast to cytokinin, ABA synthesis will be induced in response to water loss, which represents a protective role in response to adverse conditions *via* arresting seed germination and postgermination growth (Lopez-Molina et al., 2002), modulating stomata closure (Zhang et al., 1987) as well as regulating the expression of stress-responsive genes (Zhu, 2002). An earlier study found that trans-zeatin riboside decreases significantly in sunflower plants upon exposure to drought stress, while the endogenous ABA content was increased (Hansen and Dörffling, 2003). Jeon et al. (2010) reported that AHK2, AHK3, and A-type ARRs act as negative regulators in cold stress signaling pathway *via* inhibiting ABA response. It is illustrated that both the *ahk2 ahk3* double mutant and the *arr7* single mutant showed enhanced freezing tolerance, while they are hypersensitive to ABA. Although cytokinin and ABA antagonistically regulate many processes, the molecular mechanisms are poorly known. Recently, Huang et al. (2018) reported that the ABA-activated SnRK2s could phosphorylate the negative regulator type-A response regulator 5 and enhance its stability upon drought stress, by which amplifying the ABA-mediated stress response. This study provides insights into the molecular mechanism of how ABA and cytokinin interplay antagonistically in response to stress.

The levels of endogenous cytokinin is closely related to small molecular modification by the family 1 UDP glycosyltransferases (UGTs), which transfer sugar moieties to small acceptor molecules (Bowles et al., 2006; Lairson et al., 2008). The *Arabidopsis* UGT76C1 and UGT76C2 have been identified to finely tune the glycosylation of cytokinins, which deactivate the molecule and play crucial roles in regulating cytokinin homoestasis in plants (Hou et al., 2004). In our previous study, we revealed that *UGT76C2* is repressed by ABA, osmotic stress, and drought stress. The ectopic expression of *UGT76C2* led to sensitivity to ABA and mannitol during germination stage and tolerance to drought stress as established big plants. It is typically an ABA-dependent pathway in resisting abiotic stresses. We demonstrated that the involvement of UGT76C2 to water deprivation response can be mediated *via* cytokinin and ABA correlations in *Arabidopsis* (Li et al., 2015).

Many identified stress-regulated UGTs were also heterologously expressed in other plant species and bring special properties to the transgenic plants. For example, the ectopic expression of the *Arabidopsis UGT85A5* in transgenic tobacco enhanced salt tolerance in the plants (Sun et al., 2013). Li et al. (2017) found that a barley UDP-glucosyltransferase HvUGT13248, which modify deoxynivalenol and nivalenol, provides *Fusarium* head blight resistance in transgenic wheat. Although a number of rice UGTs are putatively stress-responsive upon examination on Genevestigator^1^, no stress-related UGTs have been functionally characterized in rice, and none of the known UGTs have been introduced into rice yet. To see what effect will a stress-related UGT bring to rice, here we transferred the *Arabidopsis* UGT76C2 into rice and further explored its role. Our results showed that AtUGT76C2 greatly enhanced the plant tolerance to drought and salt in transgenic rice in both developmental and physiological levels. This study laid a theoretical foundation that *UGT76C2* could be a promising candidate gene for cultivating saline- and drought-tolerant plants in both dicots and monocots.

## Materials and Methods

### Plant Materials, Vector Construct, and Plant Transformation

*Oryza japonica* was used in this study. Seedlings were grown on MS plates or soil under SD (10 h light/14 h dark) condition at 28°C in a growth chamber. To generate *UGT76C2* overexpression vector driven by a ubiquitin promoter, the coding region of *Arabidopsis UGT76C2* (At5g05860) was PCR-amplified and sequenced to be right. Then, the fragment was inserted into pUN1301 binary vector by *Kpn*I and *Bam*HI digestion and T_4_ ligation. Rice transformation was performed by Biorun biological company^2^.

### HPLC and LC–MS Analysis

For analyzing the reaction activity toward cytokinin, total protein was extracted from wild-type (WT), OE2, and OE41 plants. The glycosyltransferase activity assay toward trans-zeatin was carried out with 0.1 mg total protein, 1 mM trans-zeatin, 5 mM UDP-glucose, 50 mM HEPES (pH 7.0), 2.5 mM MgSO_4,_ 10 mM KCl, and 14.4 mM β-mercaptoethanol in 100 μl reaction mix. The reaction mix was incubated at 30°C for 3 h. The total protein was extracted in three biological replicates. For analyzing cytokinin glycosides produced in the plants, 7-day-old WT, *UGT76C2OE2*, and *UGT76C2OE41* were treated with 150 μmol trans-zeatin for 24 h, and the total glycosides were extracted with 80% methanol. The treatment and the total glycoside extraction were done in three biological replicates. High-performance liquid chromatography (HPLC) was performed on a Shimadzu HPLC system (Japan). Then, a 10 μl-sample was loaded onto a 5-μm C18 column (150 mm × 4.6 mm; Welch, Ultimate). A linear gradient with increasing methanol (solvent A) against 0.1% triethylamine acetate (solvent B) at a flow rate of 1 ml/min over 40 min was used. Both solutions contained 0.1% H_3_PO_4_. The peak of trans-zeatin was monitored at 245 nm. For liquid chromatography–mass spectrometry (LC–MS) analysis (Shimadzu), the methods and the mobile phases were similar to the HPLC condition. The mass spectrometer was operated in a positive electrospray ionization mode with 50 eV and a probe voltage of 5.0 kV. The dry heater was set to 180°C. Data acquisition and analysis were performed with Xcalibur software (version 2.0.6).

### Stress Assays

For calculating seed germination rates under stresses, full and same-size rice seeds were surface-sterilized in 75% ethanol for 2 min and then in 0.1% mercuric chloride solution for 3 min and rinsed three or four times. The sterilized seeds were imbibed at 28°C in the presence of water (control) and a hydroponic solution of 100 mM NaCl, 7.5% PEG8000, and 150 mM mannitol. For the ABA treatment, ABA was firstly dissolved in a small amount of ethanol and then diluted into the hydroponic solution with 2 and 5 μM ABA. The seeds were germinated in SD (10 h light/14 h dark) condition at 28°C in a growth chamber and were regarded as germinated when the radicles protrude from the seed coat over 2 mm. For each replicate, 50 seeds were calculated. The subsequent seedling growth was observed 5 days later and photographed. For drought stress treatment, watering of 2-week-old rice seedlings growing in soil was stopped until they wilted, followed by re-irrigation. After recovery, the performance of the plants in stress tolerance was observed and photographed. For salt stress, the WT and overexpression lines were irrigated with 200 mM NaCl until they show differences in stress tolerance. Each stress treatment experiment contained three biological replicates. After the treatment, the survival rate was calculated. The recovered plants were regarded as survivors.

### Activities of ROS-Scavenging Enzymes

The leaves were detached from 1-week-old rice plants after 200 mM NaCl and 15% PEG8000 treatments for 12 h, and the activities of ROS-scavenging enzymes such as APX, CAT, and SOD activities were determined according to a previously described method (Cai et al., 2015). In this assay, three independent samples were collected, and three technical replicates were done for evaluating enzyme activities.

### Diaminobenzidine and Nitrobluetetrazolium Staining, Determination of H_2_O_2_ Content

For the determination of H_2_O_2_ and superoxide accumulation under abiotic stress conditions, 4-week-old rice plants were exposed to 200 mM NaCl and 15% PEG8000 for 24 h, respectively, and then were harvested for diaminobenzidine (DAB) and nitroblue tetrazolium (NBT) staining. Briefly, the leaves were firstly infiltrated by vacuum for 1 h and then subjected to 0.1% DAB staining for 24 h (pH = 5.8), followed by incubation in the de-staining buffer (ethanol/lactic acid/glycerol = 3:1:1). DAB staining was performed according to Du et al. (2008) to determine the H_2_O_2_ content. NBT staining for superoxide detection was conducted as described by Wohlgemuth et al. (2002). At least five independent plants were subjected to staining for each line. To determine the content of H_2_O_2_, Catalase Assay Kit (S0051, Beyotime, Shanghai, China) was used according to the user’s manual. In brief, the 4-week-old rice plants were treated under salt and drought stress for 24 h, and 0.1 g of tissue was harvested and homogenized with reagents in the kit. Being catalyzed by the catalase, the accumulated H_2_O_2_ was changed into a red product that can be determined by a spectrophotometer at A_520_. In the assay, H_2_O_2_ was determined at three biological replicates.

### Determination of Cytokinin Content

To determine the cytokinin content, 0.1 g of fresh leaves of different lines were weighed, and for each line, three independent samples were collected as three biological replicates. The leaf tissues were homogenized in 900 μl phosphate-buffered saline (PBS; pH 7.2–7.4) and centrifuged for 20 min at the speed of 3,000 rpm, and then the supernatant was collected. For the quantification of cytokinin, ELISA Kit (MM-3259201)^3^ was used. The concentration of cytokinin in the samples is determined by a microplate reader (Tecan, InfiniteTMM200 PRO).

### Determination of Proline and Soluble Sugar Content

To determine the content of proline and soluble sugar, 4-week-old rice plants growing under normal condition were treated with 200 mM NaCl and 15% PEG8000 for 24 h, respectively, and the untreated plants served as control. For each line, three independent samples with 0.1 g harvested leaf tissues were collected. The samples were homogenized in 3% aqueous sulphosalicylic acid and centrifuged. The supernatant was collected, and equal volumes of glacial acetic acid and ninhydrin were added. The content of proline was determined at 520 nm (Nakazawa et al., 1982). To examine the content of soluble sugar, 0.1 g of harvested leaves was crushed into powder in liquid nitrogen and homogenized in 80% ethanol, followed by incubation at 95°C for 1 h. The supernatant was collected by centrifugation, dried at 80°C for 2 h, and then dissolved in 10 ml distilled water. Quantification of soluble sugar was performed as described by Li et al. (2004).

### Calculations on Ion Leakage, Water Loss, and Water Content of Detached Leaves

Upon exposure to 200 mM NaCl for 7 days, rice leaves were collected from 2-week-old rice seedlings to measure the electrolyte leakage as described by Tang et al. (2017). For calculating water loss, fresh leaves detached from 3-week-old plants were firstly weighed (FW) and then dried naturally in open air. The drying leaves were weighed (DW) every 30 min. Water loss was calculated as (FW − DW)/time ^∗^ FW. For each assay, three biological replicates were performed.

### Observations of Stomatal Opening by Scanning Electron Microscopy

Leaves of 3-week-old *UGT76C2* transgenic rice and WT plants were detached and kept in air for 2 h to allow water loss and stimulate stomatal closure; then, the leaf tissues were fixed in 2.5% glutaraldehyde at 4°C for 12 h and then washed five times with 0.1 mol/L phosphate buffer, followed by dehydration with gradient ethanol. The samples were bonded with a drying instrument on the HCP-2 critical point, followed by spraying gold with an IB-v ion sputtering device and photographed with a scanning electron microscope (FEI Quanta250 FEG). For each line, at least 100 stomata were observed.

### Determination of ABA Content

Leaves of 3-week-old *UGT76C2* transgenic rice and WT plants were detached and kept in air for 2 h. For each line, three independent samples were collected, at approximately 50 mg leaf tissue for each sample. The samples were rapidly frozen with liquid nitrogen, followed by homogenization in PBS (pH 7.4). The samples were centrifuged for 20 min, and the supernatant was collected carefully for determination. The ABA concentration was assayed according to the instructions provided by the plant hormone abscisic acid ELISA Kit (Shanghai Fusheng Industrial Co., Ltd., catalog number: A112641-96T).

### Quantitative and Semi-Quantitative RT-PCR

For quantitative and semi-quantitative RT-PCR, total RNA was extracted from rice samples with Trizol reagent (Vazyme), and 5 μg RNA was reverse-transcribed with the PrimeScript RT reagent kit with gDNA Eraser (Vazyme) according to the supplier’s manual. Real-time PCR was done with a real-time thermal cycling system (Bio-Rad). SYBR-Green was used to detect gene abundances. Each reaction was done with three biological replicates. Data were analyzed using Bio-Rad CFX Manager software. For evaluating *UGT76C2* expression in transgenic rice, semi-quantitative RT-PCR was employed. The synthesized cDNA was diluted five times (1:5), and 2 μl was used for analyzing the transcript level. *OsActin1* was used as an internal reference gene. PCR reactions were performed in 25 μl of total reaction volume, with 25 cycles for amplifying *OsActin1* and 32 cycles for amplifying *UGT76C2*. The primers for the RT-PCR assay are included in Supplementary Table S1.

### Statistical Analysis

Student’s *t*-test with one-tailed test was performed to compare the performance of WT and *UGT76C2* transgenic lines. For each assay and experiment, data from at least three biological replicates were subjected to statistical analysis. The minimum significance levels were set at ^∗^*p* < 0.05 and ^∗∗^*p* < 0.01.

## Results

### Overexpression of the *Arabidopsis UGT76C2* in Rice Generates More Cytokinin Glycosides

**FIGURE 1:**
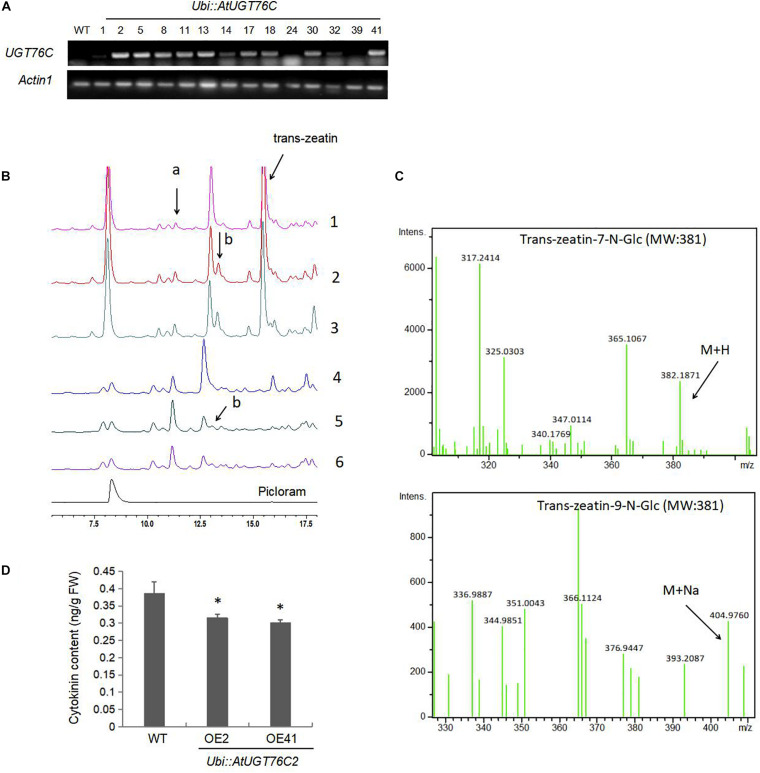
The Arabidopsis UGT76C2 glycosylates cytokinin in transgenic rice. **(A)** RT-PCR analysis of the mRNA levels of *UGT76C2* in transgenic rice plants. **(B)** Trans-zeatin catalyzing activity of the total proteins extracted from WT (1), OE2 (2), OE41 (3), and analysis of total glycosides extracted from WT (4), OE2 (5) and OE41 (6) after 150 n mol trans-zeatin treatment for 24 h. The product (a) indicates trans-zeatin-7-N-Glc, (b) indicates trans-zeatin-9-N-glc. The experiments were conducted in three biological replicates. **(C)** LC-MS confirmation trans-zeatin-7-N-Glc and trans-zeatin-9-N-glc. **(D)** The content of cytokinin in 2-week-old seedlings of WT and *UGT76C2* transgenic rice. Bars show standard deviations of three biological replicates. Asterisks indicate significant differences between the wild type and the overexpression lines evaluated with Student’s *t*-test (^∗^*P* < 0.05).

To determine the role of the *Arabidopsis* UGT76C2 in rice, transgenic plants overexpressing *AtUGT76C2* under the control of the maize ubiquitin (ubi) promoter were generated, and the homozygous lines were selected with hygromycin. qRT-PCR analysis indicated that the transcripts of *AtUGT76C2* could be detected in transgenic plants, but not in WT plants (Figure 1A). The *UGT76C2* overexpression lines OE2 and OE41 with relatively higher *UGT76C2* abundances were chosen for all assays in this study. To determine the role of UGT76C2 in catalyzing cytokinins in rice, firstly, we purified the total protein from WT, OE2, and OE41 plants and assessed their activity in catalyzing trans-zeatin. It was found that more trans-zeatin-7-N-Glc and trans-zeatin-9-N-Glc were produced after incubation with total protein purified from OE2 and OE41 than that from WT (Figures 1B1–3). The identification of 7 and 9-N-Glc was based on their retention time as reported by Hou et al. (2004) and by LC–MS confirmation (Figure 1C). To further investigate UGT76C2 activity toward trans-zeatin in rice, 7-day-old WT, *UGT76C2*OE2, and *UGT76C2*OE41 seedlings were treated with 150 μmol trans-zeatin for 24 h. Then, total glycosides were extracted from the three plants and subjected to HPLC and LC–MS analysis. Consistent with a previous study (Hou et al., 2004), more 7-N-Glc was generated than 9-N-Glc in plants (Figures 1B4–6), which further demonstrates that UGT76C2 has a higher activity toward 7-N of trans-zeatin. However, for the total protein-catalyzed reaction *in vitro*, it seems that more 9-N-Glc was produced than 7-N-Glc (Figures 1B1–3). It is likely that UGT76C2 activity is affected by other proteins in the mix. These results showed that *UGT76C2* overexpression in rice could catalyze the glycosylation of cytokinin. In addition, we also evaluated the endogenous cytokinin content in the three plants and found that it decreased in the two UGT76C2 overexpression lines (Figure 1D), demonstrating that UGT76C2 affected cytokinin homeostasis in transgenic rice.

### Overexpression of the *AtUGT76C2* in Rice Leads to Sensitivity to Abiotic Stresses and ABA at Germination and Postgermination Stages

**FIGURE 2:**
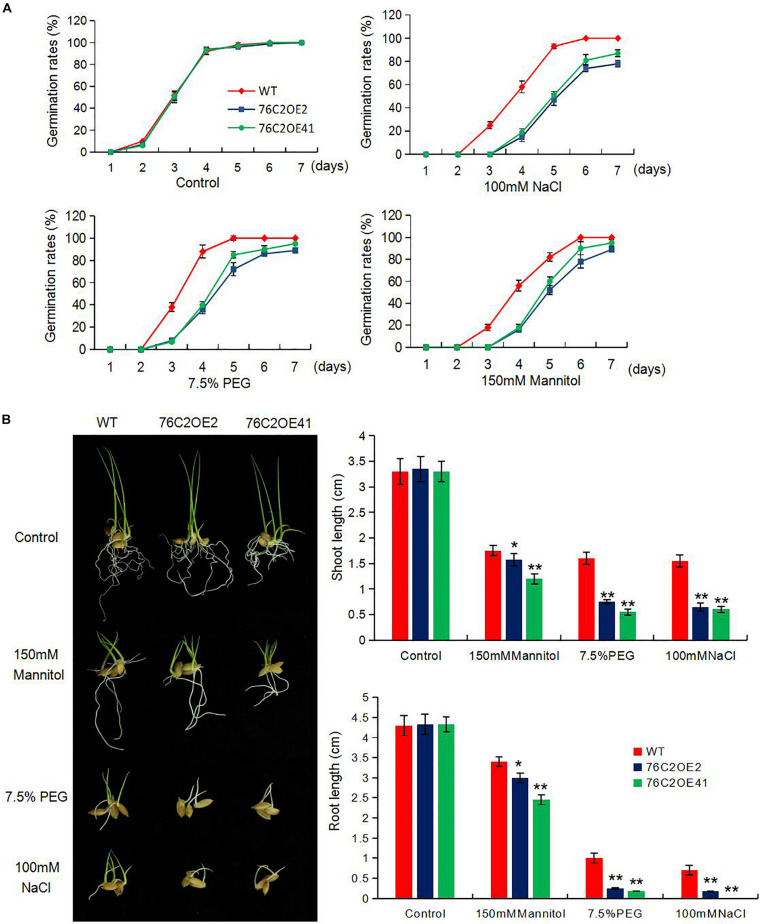
Overexpression of AtUGT76C2 increased the plant sensitivity to abiotic stresses during germination and post-germination growth. **(A)** Rice seeds were imbibed at 28°C in the presence of water (Control) and hydroponic solution of 100 mM NaCI, 7.5% PEG and 150 mM mannitol. Germination (based on radicles>2 mm) of at least 50 seeds were recorded for each biological replicates, and three biological replicates were done for calculating germination rates **(B)**. Post-germination growth of the seedlings under abiotic stresses, and shoot and root lengths were recorded. Bars show standard deviations of three independent replicates. Asterisks indicate significant differences between the wild type and the overexpression lines evaluated with Student’s *t*-test (*P < 0.05, **P < 0.01).

**FIGURE 3:**
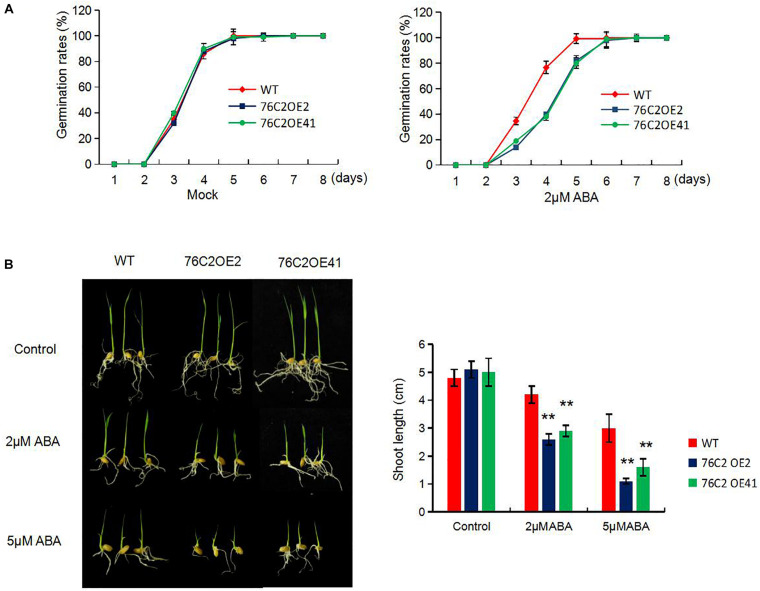
Overexpression of *AtUGT76C2* increased the plant sensitivity to abscisic acid (ABA) during germination and post-germination growth. **(A)** Rice seeds were imbibed at 28°C in hydroponic solution with 2 μM ABA (dissolved with little ethonal) and without ABA (Mock, with equal amount of ethonal). Germination (based on radicles>2 mm) of at least 50 seeds were recorded for each biological replicates, and three biological replicates were done for calculating germination rates **(B)**. Post-germination growth of the seedlings under ABA, and shoot lengths were recorded. Bars show standard deviations of three independent replicates. Asterisks indicate significant differences between the wild type and the overexpression lines evaluated with Student’s *t*-test (**P < 0.01).

To see the performance of the *UGT76C2* transgenic lines in response to abiotic stresses, seeds of wild type, *UGT76C2*OE2, and *UGT76C2*OE41 were exposed to 100 mM NaCl, 150 mM mannitol, and 7.5% PEG for germination, and the germination rates were recorded in the following 8 days. As illustrated in Figure 2A, OE2 and OE41 showed lower germination rates compared to WT under these adverse conditions. The subsequent growth of OE2 and OE41 seedlings was also more affected under abiotic stresses. The two OE lines were observed to have shorter shoots and roots than that of WT when kept growing under mannitol, NaCl, and PEG conditions for 1 week more after germination. Especially the shoot growth of *76C2*OE lines is significantly inhibited under PEG and NaCl treatments (Figure 2B). Next, we also examined the response of *UGT76C2* overexpression lines upon ABA treatment in terms of seed germination and shoot growth. It was observed that OE2 and OE41 exhibited lower germination rates as well as slower shoot growth (Figure 3).

### Expression of *AtUGT76C2* Improves Stress Adaptation and Enhances Root Growth at Seedling Stage

**FIGURE 4:**
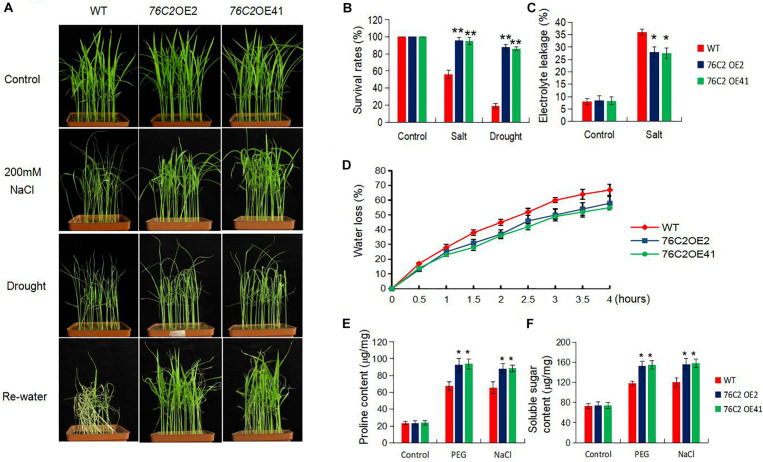
Overexpression of *AtUGT76C2* in rice enhanced salt and drought tolerance in the adult transgenic plants. **(A)** For salt treatment, 2-week-old rice seedlings were exposed to 200 mM NaCI for 7 days. For drought treatment, the plants were dried for 1 week, and then allowed for recovery for 7 days. **(B)** Survival rates of the transgenic and the control rice after salt and drought treatment. **(C)** Electrolyte leakage of the plants after salt treatment. **(D)** Water loss rate of detached leaves during 4 h. Proline **(E)** and soluble sugar **(F)** content under PEG and NaCI treatments. Each assay was done at three independent biological replicates. Asterisks indicate significant differences between the wild type and the overexpression lines evaluated with Student’s *t*-test (*P < 0.05, **P < 0.01).

To see the performance of *AtUGT76C2* overexpression plants under salt stress, the soil-growing 2-week-old WT, *UGT76C2*OE2, and *UGT76C2*OE41 seedlings were exposed to 200 mM NaCl for 2 weeks, and then the WT plants became withered, while the transgenic lines were more tolerant to salt stress (Figure 4A). Their tolerance to salt stress was also assessed in terms of survival rate and electrolyte leakage. The survival rates of WT, OE2, and OE41 were 53.6, 93.3, and 92.1%, respectively (Figure 4B), and the two transgenic lines OE2 and OE41 exhibited less electrolyte leakage under salt stress than WT, indicating that less damage is caused in the *UGT76C2* overexpression plants (Figure 4C). For drought stress treatment, 2-week-old WT, OE2, and OE41 were dried for 7 days and then re-irrigated. It was observed that most of the WT plants wilted (Figure 4A), while the two OE lines were still vigorous and showed higher survival rates, 86.2 for *76C2*OE2 and 85.3% for 76C2OE41, respectively. However, the survival rate of WT plants was only 18.8% (Figure 4B). To make a further investigation, we also detected the water loss rates of the detached leaves from the three plants in 4 h. The result indicates that the two transgenic rice lines exhibited lower water loss than that of WT. After 4 h, the water loss rates of WT, OE2, and OE41 were 67.1, 58.0, and 55.2%, respectively (Figure 4D). Additionally, the proline and soluble sugars, which serve as osmotic regulators, were also accumulated more in OE2 and OE41 plants (Figures 4E,F). Above all, from both visual phenotype and assessment of physiological indexes, drought and salt stress tolerance is greatly improved in *UGT76C2*OE rice plants.

**FIGURE 5:**
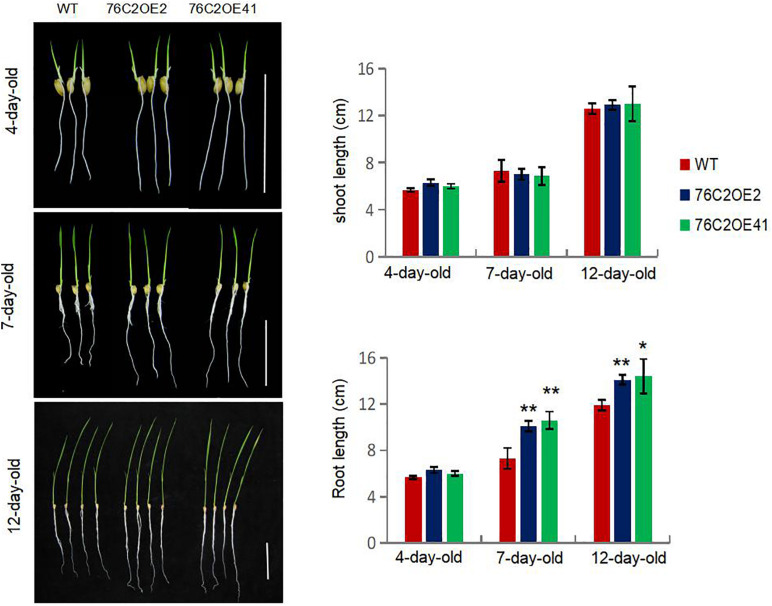
Overexpression of *AtUGT76C2* in rice enhanced root growth (bar = 5 cm). Bars show standard deviations of at least 10 seedlings. Asterisks indicate significant differences between wild type and the overexpression lines evaluated with Student’s *t*-test (*P < 0.05, **P < 0.01).

Interestingly, it was also observed that, with the growth of the rice seedlings, root growth was enhanced upon *UGT76C2* overexpression in the transgenic lines. For seedlings as young as 4 days old, they showed little difference in root length. However, for 7, 10, and 15-days-old seedlings, the roots of the UGT76C2 overexpression lines were obviously longer than those of WT (Figure 5), which also contributes to the drought and salt stress tolerance of the UGT76C2 overexpression lines.

### Overexpression of *AtUGT76C2* in Rice Reduced Stomatal Opening in Response to Drought Stress

**FIGURE 6:**
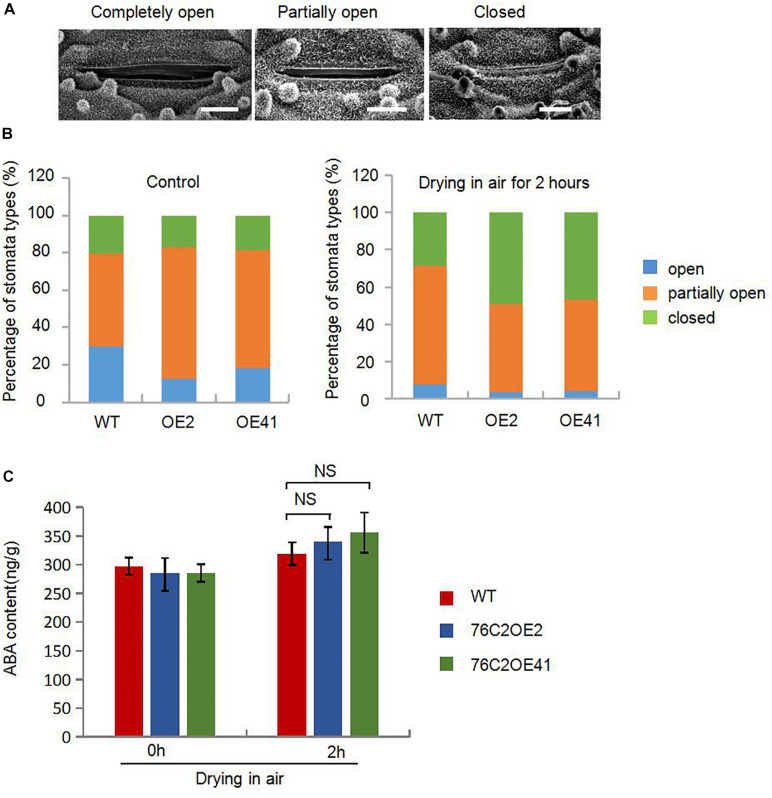
AtUGT76C2 enhanced the plant stomata closure in rice in response to water loss. **(A)** The opening of stomata observed with a scanning electron microscope. Bar = 5 urn. **(B)** The proportion of three stomata openings in WT and *AtUGT76C2* overexpression lines (*n*> 100). **(C)** ABA contents in WT and *AtUGT76C2* overexpression lines which were measured at three biological replicates.

When suffering from drought stress, plants will soon reduce the transpiration rate and water loss, for example, by controlling stomata movement. Therefore, we compared the stomatal opening of 4-week-old rice leaves of WT, *UGT76C2*OE2, and *UGT76C2*OE41. The degrees of stomatal opening are shown in Figure 6A. Under the non-treatment condition, the proportion of closed stomata showed a little difference in WT and transgenic plants at 20.2, 17, and 18.7% for WT, OE2, and OE41, respectively. However, the proportion of completely open stomata was more in WT than in OE plants and that of partially open stomata was more in OE than in WT. After exposing the detached leaves in open air for 2 h, the number of closed stomata increased and that of completely open stomata substantially decreased. More completely closed stomata and less completely/partially open stomata were observed in OE2 and OE41 leaves compared with WT. The proportion of completely closed stomata accounts for 28.4, 48.8, and 46.5% for WT, OE2, and OE41, respectively. The completely open stomata for WT, OE2, and OE41 plants account for 64, 47.2, and 49.6%, respectively, and the partially open stomata were 7.6, 3.9, and 4% for WT, OE2, and OE41 (Figure 6B). These results suggested that the elevated water saving capacity and the drought resistance in *AtUGT76C2* transgenic rice were largely due to reduced stomatal opening.

Stomatal movement is known to be closely related to ABA level or ABA signaling. Here we determined the ABA contents in WT and *UGT76C2OE* plants under normal and drought stress conditions. It was observed that the endogenous ABA levels in both *AtUGT76C2* overexpression and WT seedlings were more accumulated in response to drought stress; however, we did not see any significant differences in ABA levels between transgenic and WT seedlings under both normal and drought stress conditions (Figure 6C). These results imply that *UGT76C2* overexpression might have affected ABA signaling instead of ABA synthesis.

### Overexpression of *AtUGT76C2* Enhanced ROS Scavenging in Transgenic Plants

**FIGURE 7:**
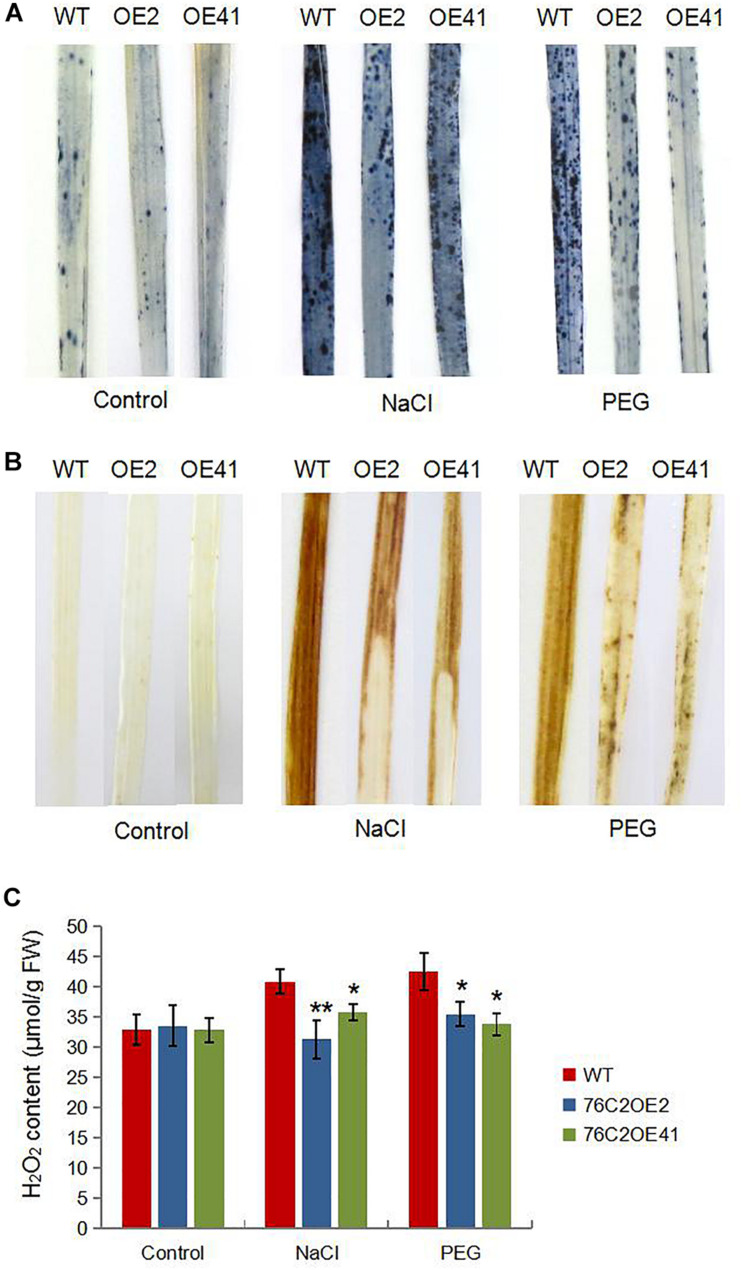
ROS production in the transgenic plants in response to salt and drought conditions. Four-week-old WT and *AtUGT76C2* overexpression rice plants were subjected to 200 mM NaCI and 15% PEG8000 treatments for 12 h, respectively. Then rice leaves were subjected to NBT **(A)** and DAB staining **(B)**, and H_2_O_2_ contents were quantitatively measured **(C)**. For each staining and H_2_O_2_ determination, leaves were harvested from at least five independent plants, and the pictures were representative of most samples. Asterisks indicate significant differences between wild type and the overexpression lines evaluated with Student’s *t*-test (*P < 0.05, **P < 0.01).

**FIGURE 8:**
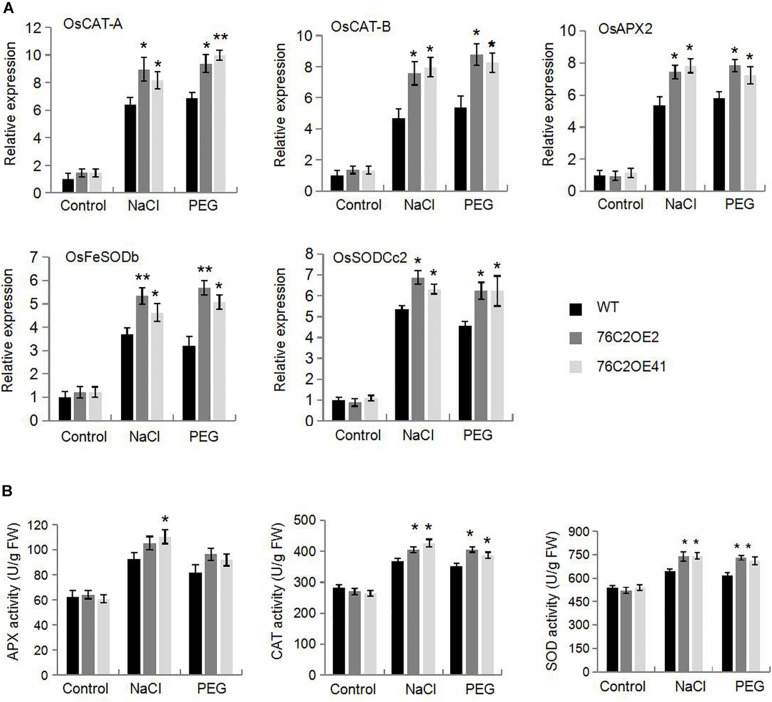
Overexpression of *AtUGT76C2* enhanced ROS scavenging in the transgenic plants. Four-week-old WT and *AtUGT76C2* overexpression rice plants were subjected to 200 mM NaCI and 250 mM PEG treatments for 12 h, respectively. Then the transcript levels **(A)** and activities of ROS scavenging enzymes **(B)** were determined. These experiments were done at three independent biological replicates. Asterisks indicate significant differences between the wild type and the overexpression lines evaluated with Student’s *t*-test (*P < 0.05, **P < 0.01).

It is known to all that stresses cause an accumulation of reactive oxygen species (ROS) that can damage the membrane systems. Here, we also investigated ROS levels in *UGT76C2OE* and WT plant under stress condition. DAB and NBT staining indicated that ROS accumulated more in WT than in *UGT76C2* transgenic lines (Figure 7). To explain the differential ROS production, we firstly examined the expression of genes encoding ROS scavenging enzymes, such as catalase isozyme A (CAT-A), catalase isozyme B (CAT-B), ascorbate peroxidase 2 (APX2), iron-superoxide dismutase b (Fe-SODb), and copper/zinc-superoxide dismutase (SODCc2). Quantitative RT-PCR showed that all the five genes were upregulated by salinity and drought and were more upregulated in the *AtUGT76C2* transgenic lines (Figure 8A). Besides, we also examined the antioxidant enzyme activities of CAT, APX and SOD in WT and transgenic plant under normal and stress conditions, and found that they were higher in transgenic lines than in WT in response to abiotic stresses, whereas showed no difference under control condition (Figure 8B).

### Expressions of Stress-Related Genes Were Affected Upon *UGT76C2* Overexpression in Rice

**FIGURE 9:**
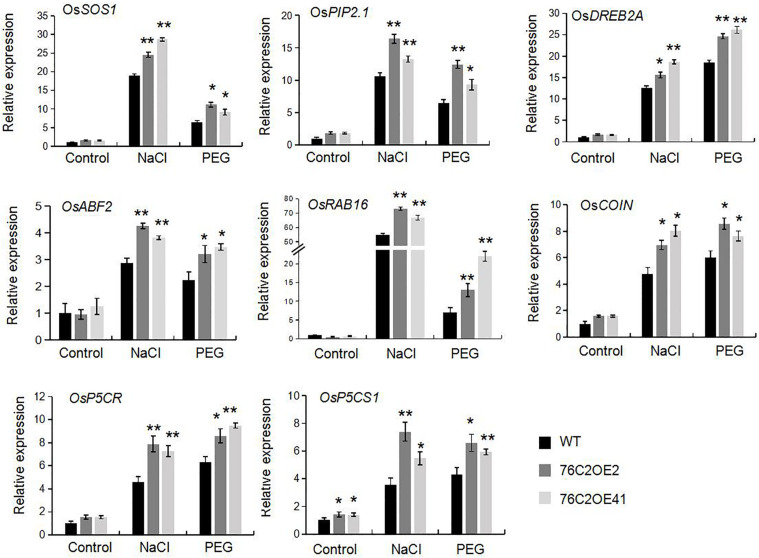
Expression of abiotic stress related genes in wild type, *UGT76C2* overexpression plants in response to stresses. The experiments included three biological replicates, each with three technical replicates. Asterisks indicate significant differences between the wild type and the overexpression lines evaluated with Student’s *t*-test (*P< 0.05, **P < 0.01).

To investigate the potential molecular pathways affected by UGT76C2 in regulating stress tolerance, we then monitored the expressions of eight abiotic stress-related genes including *OsSOS1*, *OsPIP2.1*, *OsDREB2A*, *OsCOIN*, *OsABF2*, *OsRAB16*, *OsP5CR*, and *OsP5CS1*. Two-week-old wild type and *UGT76C2*OE plants were exposed to NaCl and PEG, respectively, for 12 h, and qPCR was performed. The results showed that all these genes were induced at different degrees by salt and drought and were more highly expressed in the overexpression lines compared with that in WT plants (Figure 9). For instance, in response to salt stress, the expression of *OsSOS1*, *OsPIP2.1*, and *OsRAB16* were significantly induced, more than 15-folds in transgenic lines, while less induced in WT plants. Upon exposure to PEG, *DREB2A* was substantially elevated in the two overexpression lines, up to 25-fold, while less than 20-fold in WT plants. The differential expression of these stress-regulated genes also accounted for the stress tolerance of *76C2*OE plants.

## Discussion

Rice is the most consumed staple food in the world that feeds more than half of the world’s population. However, with the deterioration of the current global environment and uneven precipitation, the area of soil drying is gradually increasing. As a semi-aquatic crop, rice is greatly threatened by drought and salt stresses. In order to cultivate rice varieties with good traits, it is important to explore candidate genes that can be applied into rice breeding. To cope with adverse environmental stimuli, scientists are devoted to identifying stress-responsive genes and generate stress-adaptive crops by molecular breeding or gene engineering. Adverse environmental factors affect many aspects of plants but will finally lead to disturbance of the metabolic process that closely associated with plant growth and development. UDP-glycosyltransferases, as the manager of cellular homeostasis in plants (Lim and Bowles, 2004; Bowles et al., 2005), likely provide new strategies in cultivating stress-resistant crops.

In this study, we introduced the Arabidopsis UGT76C2 into rice, which affects cytokinin homeostasis (Figure 1) and enhances stress tolerance in the transgenic rice (Figure 4). We demonstrate that UGT76C2 contributes to stress tolerance in several aspects. First, overexpression of *UGT76C2* decreased the level of cytokinins (Figure 1D), which is generally regarded as a negative factor in plant abiotic responses (Nishiyama et al., 2011). *UGT76C2* overexpression also enhanced root growth in the transgenic lines (Figure 5). This finding is consistent with several former studies indicating the role of cytokinin in modulating root development (Zalabák et al., 2013). For instance, overexpression of *CKX* in barley led to cytokinin deficiency, which enlarged the root system and increased root biomass (Zalewski et al., 2010). Pineda Rodó et al. (2008) found that constitutive expression of a zeatin-O-glycosyltransferase in maize resulted in more branched roots with greater biomass. A former study in our group also reported that overexpression of *UGT76C2* enhanced the lateral root density in Arabidopsis (Wang et al., 2011). A strong root system is a critical trait in adapting to water deficit. A recent study found that the ZmPTF1 transcription factor enhanced drought tolerance by promoting root growth (Li et al., 2019). Additionally, according to Li et al.’s (2018), overexpression of *RCc3* in rice increased the root system and enhanced salt tolerance. Thus, it is believed that enhanced root growth in *UGT76C2* overexpression lines greatly contribute to plant tolerance to drought and salt.

It is well known that abiotic stresses cause an excess production of reactive oxygen species. Cellular ROS are usually maintained at a relatively low level through a wide range of scavenging and detoxification mechanisms. More and more studies have identified the connection between cytokinin and oxidative stress. Wang et al. (2015) showed that the overproduction of cytokinin by overexpressing *AtIPT8* increased ROS production and decreased the CAT and POD activity in response to salt stress, which thus declined salt stress resistance. Additionally, Zwack and Rashotte (2015) showed that cytokinin response factor 6 functions in mediating oxidative stress response *via* attenuating cytokinin signaling. In our study, we found that overexpression of *AtUGT76C2* in rice, which decreased cytokinin level, resulted in less ROS accumulation under drought and salt stresses. Furthermore, the transcripts of ROS-scavenging enzymes such as CAT, SOD, and POD were upregulated, and their enzyme activities were also enhanced upon UGT76C2 overexpression (Figure 8). These findings further suggest that cytokinin plays a negative role for the plant to cope with oxidative stress, which is in agreement with former studies (Wang et al., 2015; Zwack and Rashotte, 2015).

We also found that the ectopic expression of *UGT76C2* impeded seed germination and postgermination growth under drought and salt stress (Figures 2, 3) while improving stress tolerance in transgenic rice seedlings (Figure 4), suggesting that the role of *AtUGT76C2* is likely related to ABA signaling. ABA plays pivotal roles in response to abiotic stresses. When the plants are challenged by water deficit, ABA is soon synthesized and triggers the expression of many ABA-dependent genes in coping with stresses. During the germination and the postgermination stages, ABA helps to arrest the germination and the postgermination growth, which is actually a protective mechanism to young seedlings (Lopez-Molina et al., 2002). After the plants grow up into adult plants, ABA on one side contributes to induce stomatal closure in response to drought stress and on the other side activates the expression of some stress-responsive genes to cope with adverse conditions. Consistently, in our study, more closed stomata were observed in *UGT76C2*-overexpressed rice than in WT plants when confronting drought stress (Figure 6B). Although the ABA level was not affected in *UGT76C2*OE plants in response to dehydration (Figure 6C), it is speculated that ABA signaling is enhanced. This point could also be approved by the upregulation of ABA signaling or responsive genes in *UGT76C2*OE plants. For instance, OsABF2 and OsRAB16 are positive regulators in ABA signaling pathway (Lu et al., 2009; Hossain et al., 2010), and both genes were more upregulated upon *UGT76C2* overexpression in response to drought and salt. *OsPIP2.1* is strongly induced by ABA, and *PIP2.1* activation was required for stomatal closure in response to ABA treatment in *Arabidopsis* (Rodrigues et al., 2017; Ding et al., 2019). *OsCOIN* is induced by ABA as well as cold, salt, and drought stresses. The overexpression of *OsCOIN* could increase tolerance to chilling, salt, and drought and enhance proline level in rice (Liu et al., 2007). The expression of the proline biosynthesis genes *OsP5CS1* and *OsP5CR*, which are responsive to osmotic and salt stresses and ABA (Hu et al., 1992; Sripinyowanich et al., 2013), was also upregulated in *UGT76C2* overexpressors under salt and drought (Figure 9).

UGT76C2 recognizes all cytokinins and modifies the hormones at the N7 and N9 positions (Hou et al., 2004). The cytokinin glucosides are generally regarded as biologically inactive CK forms in CK signaling (Hothorn et al., 2011; Lomin et al., 2015). Interestingly, there is evidence indicating that the N-linked (N6 and/or N9) cytokinin glycosides show antisenescent and antioxidant activity (Hönig et al., 2018). The effects might be explained by the presence of electronegative atoms, which are near the N6 and/or N9 atoms of purine (Hönig et al., 2018). A recent study also reported that some types of N-linked CK glucosides, such as transzeatin (tZ) N7- and N9-glucosides, efficiently release free CK bases that are probably responsible for the biological activities (Hoyerová and Hošek, 2020). Additionally, in a latest study, Hallmark et al. (2020) reported that the exogenous application of trans-Zeatin-N-glucosides could lead to CK response. All these findings updated our understanding in the role of cytokinin glycosides. It can be inferred that the heterologous expression of *Arabidopsis UGT76C2* into rice might also cause some other effects in the biochemical and molecular levels that are beyond our study here. Furthermore, cytokinin signaling under abiotic stress is acting as an inter-cellular communication network, which is essential to crosstalk with other types of phytohormones except ABA. It was demonstrated that cytokinin and auxin play complementary actions in regulating a series of plant developmental processes, which also work together in plant response to stresses (Bielach et al., 2017). A link also exists between cytokinin and SA signaling pathway *via* ARR2 and TGA3 in stress response (Verma et al., 2016). From these points of view, cytokinin-regulated stress response is far more complicated than we have revealed here. The detailed mechanisms remain elusive and require further investigation for more insights into cytokinin involvement in plant defense systems. Anyway, however, we reveal that the overexpression of *UGT76C2* in rice brings good traits in coping with stresses, which lays a theoretical foundation that *UGT76C2* could serve as a promising candidate gene for cultivating saline- and drought-tolerant crops.

## Data Availability Statement

The raw data supporting the conclusions of this article will be made available by the authors, without undue reservation.

## Author Contributions

YL, FL, and PL carried out the experiments. YL, TW, and CZ analyzed the data. YL and BH conceived and designed the research. YL and FL wrote the manuscript. All authors read and approved the final manuscript.

## Conflict of Interest

The authors declare that the research was conducted in the absence of any commercial or financial relationships that could be construed as a potential conflict of interest.

## Footnotes

1 https://genevestigator.com/

2 http://www.biorun.net/

3 http://www.mmbio.cn/
