# Supplementary material for: An Arabidopsis Cytokinin-Modifying Glycosyltransferase UGT76C2 Improves Drought and Salt Tolerance in Rice
Source: Front Plant Sci. 2020 Nov 5;11:560696. doi: 10.3389/fpls.2020.560696 (PMC7674613; doi:10.3389/fpls.2020.560696)
Supplement: Supplementary file 1 [file Data_Sheet_1.docx]

Supplementary Material

Supplementary Tables

| Gene | Forward (5’-3’) | Reverse (5’-3’) |
| --- | --- | --- |
| **Primers for gene cloning** | | |
| *AtUGT76C2* | GGATCCATGGAGGAGAAGAGAAATGG | GGTACCTTACAACAATAGTATATGATTAGCT |
| **Primers for qRT-PCR analysis** | | |
| *OsActin1* | GACCTTGCTGGGCGTGAT | GTCATAGTCCAGGGCGATGT |
| *OsAPX2* | TTCAGCTTTCGTTTGTGCGG | ACCACTCGCAATCCAACGAT |
| *OsCAT-A* | TTGGGGGTGAAGATTGCGAA | CGACAACAGAAGATGCGTGC |
| *OsCAT-B* | GCTTGCACAGTTTGACAGGG | CGACTGTGGAGAACCGAACA |
| *OsFeSODb* | ACAACGGCAACCCATTACCA | CAGCCAGACCCCAAAAGTGA |
| *OsSODCc2* | CCTAAACACGCAGCGACAAC | CCAGAGACACTTCCCGTCAC |
| *OsSOS1* | TCTGCAAAGGAGTGCGTCAT | TCATGCTCCCGTACATGCTC |
| *OsDREB2A* | ATGTATGGTCCCACAGCACG | ACAACACAGCTGGCCCATTA |
| *OsPIP1.1* | ACTACTAAGCTCATCGCCGC | GGTTCCCAAAGGTCCACACT |
| *OsPIP2.1* | CTAAGCTAGGTCGGGCATGG | TGGTGCTTGTACCCGATCAC |
| *OsP5CR* | TCCTCGGGATCTTGCACTTG | CACGGAATGCACCCTTCTCA |
| *OsP5CS1* | AAGTTCCGGGAGCAACTCAC | AGTTCCAGTGCCAACAGTCC |
| OsCOIN | TCAAAGCAGCAAATGCACCC | CATGGCCATCAATGAAGCGG |
| OsABI2 | GATGGTCTCTGGGACGTCAT | CTTCGACAAGCACTCAGCAG |
| OsRab16 | ATGAGGGAGGAGCACAAGAC | TTGATCCCCTTCTTCCTCCT |
| *AtUGT76C2* | CGATCCGTTCTTACATGCTGTAGTC | GCAATCTCCAAGAACTCTGTTTCC |

## Supplementary Figures


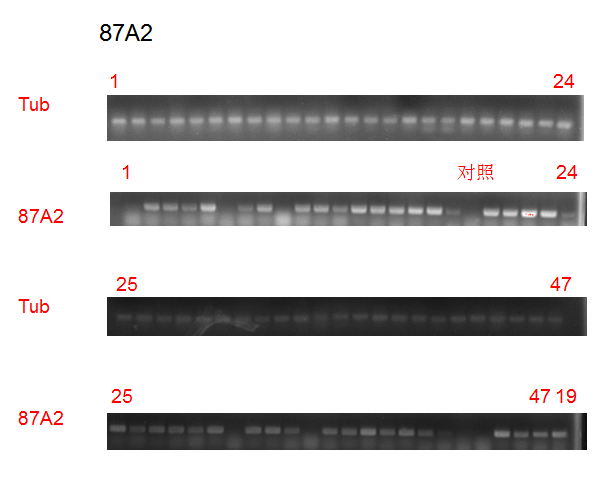

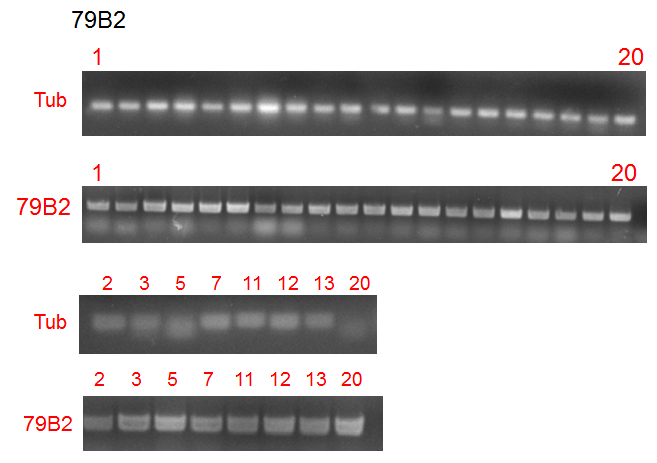


*UGT76C2*

*Actin1*

WT 1 2 5 8 11 13 14 17 18 24 30 32 39 41

*Ubi::AtUGT76C2*

**Supplementary Figure 1.***UGT76C2* expression levels in transgenic rice
